# A retrospective analysis of initial ultrasonographic misdiagnosis of fibrous hamartoma of infancy at the ultrasound center of a tertiary hospital in China

**DOI:** 10.3389/fped.2026.1869269

**Published:** 2026-08-05

**Authors:** Yanmei Zhang, Fang Yang, Qi Shen

**Affiliations:** Department of Ultrasound, Anhui Provincial Children’s Hospital, Fudan University Affiliated Children’s Hospital Anhui Hospital, Hefei, Anhui, China

**Keywords:** clinical characteristics, fibrous hamartoma of infancy, misdiagnosis, superficial soft-tissue tumor, ultrasonography

## Abstract

**Background:**

Fibrous hamartoma of infancy (FHI) is a relatively rare fibroblastic or myofibroblastic tumor. FHI is highly prone to misdiagnosis prior to surgery owing to its nonspecific clinical features and imaging manifestations. We analyzed the potential factors associated with initial ultrasonographic misdiagnosis of FHI to improve the understanding and diagnostic accuracy of FHI and reduce the misdiagnosis rate among pediatricians and sonographers.

**Methods:**

We retrospectively analyzed all consecutive children with FHI at Anhui Provincial Children's Hospital between January 2012 and December 2025. The clinical characteristics, ultrasonographic features, and CT/MRI imaging findings of FHI were summarized, and potential factors associated with initial ultrasonographic misdiagnosis were analyzed.

**Results:**

Sixteen children and 17 lesions were included in this study. Fifteen children (15/16, 93.75%) were under 2 years of age, and the male-to-female ratio was approximately 3:1. Ultrasonographic examination revealed that three lesions (3/17, 17.65%) were hypoechoic, two (2/17, 11.76%) were mixed echoic, and 12 (12/17, 70.59%) were principally hyperechoic, of which two (2/17, 11.76%) manifested a “fence-like pattern”, and six (6/17, 35.29%) exhibited a “serpentine pattern”. All 16 children were initially misdiagnosed on preoperative ultrasonography. Lipoma accounted for the largest proportion (8/16, 50%), followed by infantile hemangioma (3/16, 18.75%). All 13 children who underwent CT/MRI were also misdiagnosed. Qualitative ultrasound–histopathology correlation suggested that hyperechoic lesions were mainly associated with mature adipose tissue, whereas hypoechoic lesions were mainly associated with primitive mesenchymal tissue. Follow-up data were available for five children (median, 76 months), and no local recurrence was observed.

**Conclusions:**

FHI is frequently misdiagnosed on ultrasonography. The “serpentine pattern” and “fence-like pattern” may be suggestive ultrasonographic manifestations of FHI. The proposed diagnostic flowchart, which emphasizes integrative interpretation of clinical characteristics and multimodal imaging findings, may assist pediatricians and sonographers in differentiating FHI from other soft-tissue lesions. However, a definitive diagnosis still relies on histopathological examination.

## 1 Introduction

Fibrous hamartoma of infancy (FHI) is a relatively rare fibroblastic or myofibroblastic tumor that accounts for 0.02% of all benign soft-tissue tumors. FHI is more common in male infants and children under 2 years of age, and its clinical manifestations are primarily painless subcutaneous masses (1). Most imaging examinations involve ultrasonography. Although treatment generally involves complete surgical resection, since the clinical features and imaging manifestations of FHI are mostly nonspecific, it is easily misdiagnosed prior to surgery (2, 3). Misdiagnosis can ultimately contribute to delayed treatment, errors, and patient harm; however, retrospective observational studies have revealed common factors associated with these errors (4, 5). The existing literature is typically focused on case reports or small-sample characteristic descriptions, and there is a paucity of research on the misdiagnosis patterns of FHI. In this study, we retrospectively analyzed 16 cases of FHI to summarize the clinical and ultrasonographic features as well as potential factors associated with initial ultrasonographic misdiagnosis, to improve the understanding and diagnostic accuracy of FHI and thereby reduce the misdiagnosis rate among pediatricians and sonographers.

## 2 Materials and methods

### 2.1 Study subjects

**Figure 1:**
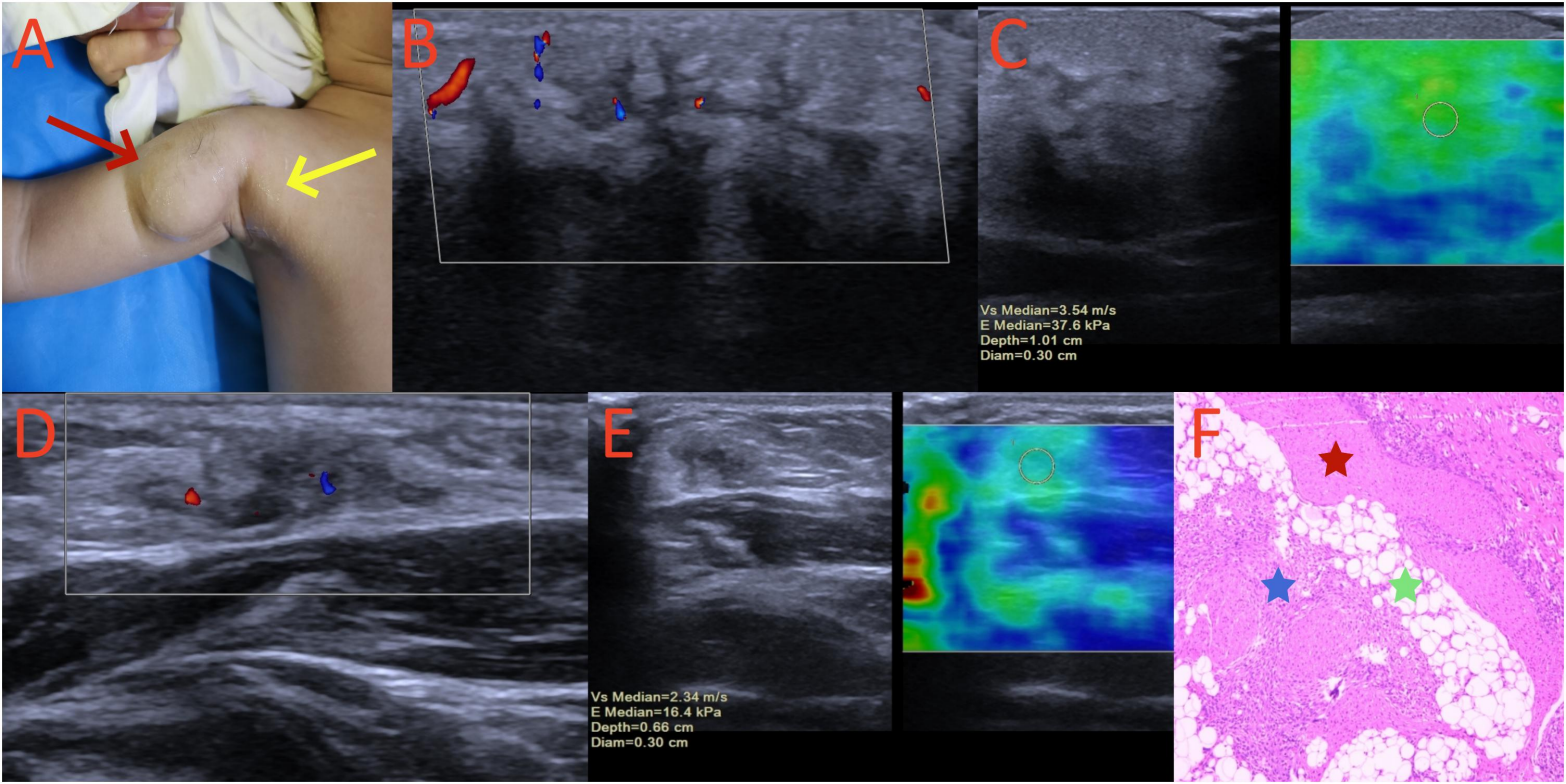
Fibrous hamartoma of infancy (FHI) in case 16. **(A)** A 19-month-old girl presenting with two lesions: one located on the right upper arm with hair growth (red arrow) and the other on the right distal clavicle (yellow arrow). **(B)** An ultrasonogram revealed a “fence-like pattern”, with alternating hyperechoic and hypoechoic areas that were perpendicular to the skin surface and dotted blood flow signals in the lesion on the right upper arm. **(C)** Ultrasonographic elastography showing that the lesion on the right upper arm has a firm texture. **(D)** Ultrasonography revealed a slightly hypoechoic lesion on the right distal clavicle, with dotted blood flow signals visible on color Doppler flow imaging (CDFI). **(E)** Ultrasonographic elastography demonstrating that the lesion on the right distal clavicle has a firm texture. **(F)** Hematoxylin and eosin (H&E) staining at 40× magnification revealed a classic triphasic morphology composed of mature adipose tissue (green asterisk), intersecting fibrous collagen tissue (red asterisk), and primitive mesenchymal tissue rich in mucoid matrix (blue asterisk).

We retrospectively reviewed the clinical records of all consecutive children confirmed to have FHI through histopathological examination at Anhui Provincial Children's Hospital between January 2012 and December 2025. All children underwent an ultrasonographic examination before surgery. This retrospective analysis was reviewed and approved by the Ethics Committee of Anhui Provincial Children's Hospital. The committee assigned no formal approval number to this retrospective study. The study was conducted in accordance with the local legislation and institutional requirements. The requirement for informed consent was waived due to the retrospective nature of the study and anonymized data analysis. Written informed consent for the publication of the clinical photograph presented in Figure 1 was obtained from the patient's legal guardian prior to submission of the manuscript. All published images were anonymized and contained no identifiable facial features.

### 2.2 Clinical data

The clinical data that we collected principally comprised the following: (1) age, sex, and medical history; (2) physical examinations for site, size, texture, boundary, mobility, relationship with surrounding tissues, tenderness, skin color and hair at the site of the mass; (3) imaging examinations [ultrasonography, computed tomography (CT), and magnetic resonance imaging (MRI)]; (4) intraoperative and postoperative histopathological findings; and (5) postoperative follow-up. For anatomical site analysis, lesions involving the breast and distal clavicular region were categorized as chest wall lesions to ensure consistent anatomical classification.

### 2.3 Ultrasonographic examination

Siemens Sequoia, Philips Epiq 5, and Mindray Resona 7 color Doppler ultrasound diagnostic instruments with broadband or frequency conversion linear array probes (probe frequency, 5–14 MHz) were used to scan the lesion surface mass from multiple sections and angles. The site, number, size, internal echo, boundary, morphology, and skin demarcation of the mass were also observed and recorded, and color Doppler flow imaging (CDFI) was used to display the internal and peripheral blood flow distribution of the mass.

### 2.4 Misdiagnosis analysis

For this study, initial ultrasonographic misdiagnosis was defined as the absence of FHI from both the primary ultrasonographic diagnosis and the differential diagnosis before surgery, with the lesion subsequently confirmed as FHI by histopathological examination.

Based on the children's medical records, clinical manifestations, preoperative imaging reports, diagnostic procedures, and final histopathological results at our hospital, we summarized and analyzed potential factors associated with initial ultrasonographic misdiagnosis and proposed a descriptive diagnostic flowchart to facilitate the diagnosis of FHI. As physician experience and technical expertise were not documented in a standardized and objective manner, these variables were not quantitatively assessed in the present study.

### 2.5 Statistical analysis

We used SPSS (version 25.0; IBM Corp, Armonk, NY, USA) for statistical analysis; however, due to the small number of cases, our analysis was primarily descriptive. Continuous variables were presented as mean and standard deviation (SD) or median and interquartile range (IQR) based on their distribution (i.e., either normal or non-normal). Categorical variables were described as frequency counts and percentages relative to the total.

## 3 Results

### 3.1 Clinical characteristics

Sixteen children with histopathologically confirmed FHI were included. All children met the criteria for initial ultrasonographic misdiagnosis. The children were aged 6–30 (with a mean of 13.6 ± 5.5) months, and 15 children were under 2 years of age (15/16, 93.75%). The male-to-female ratio was approximately 3:1. All children presented with painless local subcutaneous masses with normal skin color. Two lesions were observed in case 16, one of which showed localized hair growth. Thus, 17 lesions were included in this study and were located on the axilla (5/17, 29.41%), scrotum (4/17, 23.53%), upper arm (3/17, 17.65%), chest wall (3/17, 17.65%), neck (1/17, 5.88%), and buttock (1/17, 5.88%) (Table 1).

**Table 1:** Clinical characteristics of 16 children with FHI.

Case	Sex	Age (months)	Duration	Location	Skin color	Hair	Texture	Margin	Mobility	Tenderness
1	Male	9	4 months	Left axilla	Normal	None	Moderate	Clear	Poor	None
2	Female	10	2 months	Left axilla	Normal	None	Flexible	Unclear	Moderate	None
3	Male	14	5 months	Right upper arm	Normal	None	Flexible	Unclear	Moderate	None
4	Male	16	1 year	Right buttock	Normal	None	Flexible	Clear	Palpable	None
5	Male	12	5 months	Left axilla	Normal	None	Flexible	Clear	Palpable	None
6	Male	30	2 years	Left scrotum	Normal	None	Firm	Clear	Palpable	None
7	Female	15	7 months	Right axilla	Normal	None	Flexible	Clear	Moderate	None
8	Male	12	6 months	Right chest wall	Normal	None	Flexible	Clear	Palpable	None
9	Male	9	4 weeks	Right scrotum	Normal	None	Firm	Clear	Moderate	None
10	Male	11	6 months	Left breast	Normal	None	Firm	Clear	Moderate	None
11	Female	18	8 months	Left upper arm	Normal	None	Flexible	Clear	Palpable	None
12	Male	6	6 months	Scrotum	Normal	None	Firm	Unclear	Moderate	None
13	Male	14	5 months	Right scrotum	Normal	None	Firm	Clear	Poor	None
14	Male	11	2 months	Neck	Normal	None	Flexible	Clear	Palpable	None
15	Male	12	2 weeks	Right axilla	Normal	None	Flexible	Clear	Moderate	None
16	Female	19	1 year	Right upper arm	Normal	Yes	Flexible	Clear	Palpable	None
				Right distal clavicle	Normal	None	Flexible	Clear	Palpable	None

FHI, fibrous hamartoma of infancy.

### 3.2 Ultrasonographic findings

Seventeen lesions were examined ultrasonographically, and their longitudinal sizes ranged from 13 to 80 mm. In terms of echogenicity, three lesions (3/17, 17.65%) were hypoechoic, two (2/17, 11.76%) were mixed echoic, 12 (12/17, 70.59%) were primarily hyperechoic—with interstitial cord-like or small flake-like low echoes, two (2/17, 11.76%) of which exhibited a “fence-like pattern”, and six (6/17, 35.29%) of which exhibited a “serpentine pattern”. CDFI demonstrated no obvious blood flow signals in three lesions (3/17, 17.65%), 13 (13/17, 76.47%) showed dot-like or linear blood flow signals, and one (1/17, 5.88%) revealed rich blood flow signals (Table 2).

**Table 2:** Ultrasonographic characteristics of 17 FHI lesions.

No.	Location	Size (mm)	Echogenicity	Sonographic patterns	Margin	Morphology	Interface with skin	CDFI
1	Left axilla	33 × 30 × 12	Hyperechoic	None	Clear	Regular	Clear	Dot and stripe
2	Left anterior axilla	37 × 31 × 8	Hyperechoic	None	Unclear	Irregular	Clear	Dot
3	Right upper arm	80 × 40 × 20	Hyperechoic	None	Unclear	Irregular	Clear	Dot and stripe
4	Right buttock	70 × 50 × 20	Hyperechoic	None	Clear	Irregular	Clear	Dot
5	Left axilla	50 × 40 × 40	Mixed echoic	Serpentine	Unclear	Irregular	Clear	Dot and stripe
6	Left scrotum	20 × 19 × 9	Heterogeneous hyperechoic	Fence-like	Unclear	Irregular	Clear	None
7	Right axilla	27 × 24 × 11	Mixed echoic	Serpentine	Unclear	Irregular	Clear	Dot
8	Right chest wall	30 × 25 × 20	Hyperechoic	None	Clear	Regular	Clear	Dot
9	Right scrotum	15 × 15 × 10	Heterogeneous hyperechoic	Serpentine	Clear	Regular	Clear	Dot and stripe
10	Left breast	32 × 31 × 11	Heterogeneous hypoechoic	None	Clear	Irregular	Clear	Dot
11	Left upper arm	20 × 19 × 6	Heterogeneous hyperechoic	None	Unclear	Regular	Clear	Dot
12	Scrotum	20 × 16 × 8	Hypoechoic	None	Unclear	Irregular	Unclear	Dot and stripe
13	Right scrotum	33 × 32 × 27	Heterogeneous hyperechoic	Serpentine	Clear	Regular	Clear	None
14	Neck	30 × 26 × 16	Heterogeneous hyperechoic	Serpentine	Clear	Irregular	Clear	Abundant
15	Right axilla	16 × 14 × 10	Heterogeneous hyperechoic	Serpentine	Clear	Irregular	Clear	None
16	Right upper arm	41 × 35 × 19	Heterogeneous hyperechoic	fence-like	Clear	Irregular	Clear	Dot and stripe
17	Right distal clavicle	13 × 10 × 5	Heterogeneous hypoechoic	None	Clear	Irregular	Clear	Dot and stripe

FHI, fibrous hamartoma of infancy; CDFI, color Doppler flow imaging.

### 3.3 CT/MRI imaging findings

Thirteen children underwent cross-sectional imaging, including eight CT and five MRI examinations. One child had a negative MRI result, whereas the remaining 12 children were interpreted as having benign lesions, including three with venous malformations, three with fibromas, two with lymphatic malformations, and one each with arteriovenous malformation, lymphatic-venous malformation, infantile hemangioma, and lipoblastoma.

Retrospective analysis demonstrated that all 12 children with abnormal CT/MRI findings presented with lesions located within the dermis or subcutaneous fat layer, without involvement or invasion of the underlying musculature. The CT and MRI images were heterogeneous and mainly characterized by irregular or patchy soft-tissue density, internal septa or linear low-density area, low signal intensity on T1-weighted images, and high signal intensity on T2-weighted images. Increased signal intensity on diffusion-weighted imaging (DWI) has been observed in several cases of MRI. The enhancement patterns were variable, ranging from minimal or delayed enhancement to marked enhancement or predominantly peripheral enhancement (Table 3).

**Table 3:** Case-by-case diagnostic pathway of 16 children with FHI.

Case	Age (months)	Initial ultrasonographic diagnosis	CT/MRI performed	CT/MRI diagnosis	Key CT/MRI findings	Final histopathological diagnosis
1	9	Infantile hemangioma	CT	Lymphatic malformation	Internal linear low-density area; no significant enhancement	FHI
2	10	Lipoma	CT	Arteriovenous malformation	Linear arterial and parenchymal enhancement	FHI
3	14	Lipoma	None	/	/	FHI
4	16	Lipoma	CT	Lymphatic malformation	Patchy soft-tissue density; non-enhancing	FHI
5	12	Infantile hemangioma	CT	Fibroma	Internal septa; mild progressive enhancement	FHI
6	30	Lipoma	MRI	Negative	/	FHI
7	15	Inflammatory mass	MRI	Lymphatic-venous malformation	Low T1 and heterogeneous T2 signal intensity; predominantly peripheral enhancement	FHI
8	12	Lipoma	None	/	/	FHI
9	9	Lipoma	MRI	Fibroma	Low T1 and high T2 signal intensity; high DWI signal intensity; heterogeneous enhancement	FHI
10	11	Breast bud development	MRI	Infantile hemangioma	Low T1 and high T2 signal intensity; high DWI signal intensity; marked enhancement	FHI
11	18	Infantile hemangioma	CT	Venous malformation	Mild progressive delayed enhancement	FHI
12	6	Skin thickening	MRI	Fibroma	Low T1 and high T2 signal intensity; mildly high DWI signal intensity; strong enhancement	FHI
13	14	Hernia	CT	Lipoblastoma	Subcutaneous fat thickening; flocculent low-density area; minimal enhancement	FHI
14	11	Lipoma	CT	Venous malformation	Heterogeneous density; mild-to-moderate enhancement	FHI
15	12	Lipoma	CT	Venous malformation	Mild arterial enhancement with persistent delayed enhancement	FHI
16	19	Malignant tumor	None	/	/	FHI

FHI, fibrous hamartoma of infancy; CT, computed tomography; MRI, magnetic resonance imaging; DWI, diffusion-weighted imaging.

### 3.4 Surgical treatment, histopathological findings and follow-up

Sixteen children underwent excision surgery for surface masses and were histopathologically diagnosed with FHI, with a classic triphasic morphology composed of mature adipose tissue, intersecting fibrous collagen tissue, and primitive mesenchymal tissue rich in the mucoid matrix. Of these children, eight underwent immunohistochemical examinations, with eight positive for vimentin (Vim), seven for cluster of differentiation 34 (CD34), seven for S-100 protein (S-100), six for smooth muscle actin (SMA), one for cluster of differentiation 99 (CD99), one for cluster of differentiation 31 (CD31), one for B-cell lymphoma 2 (Bcl-2), and six for Ki-67 (proliferation index ≤ 5%).

Follow-ups were primarily conducted through outpatient visits. Follow-up data were available for five children, with a median duration of 76 months (range, 1–100 months). No local recurrence was observed among children with available follow-up data.

### 3.5 Qualitative ultrasound–histopathology correlation

A qualitative retrospective comparison between the ultrasonographic findings and available histopathological data was performed. Because immunohistochemical examinations were available for only eight children and immunophenotypes varied among cases, exhibiting some overlap and variability, immunophenotypic findings served only as supporting evidence and were summarized descriptively rather than quantitatively.

A retrospective analysis of eight pediatric cases that underwent immunohistochemical examinations suggested that hyperechoic lesions were often associated with mature adipose tissue, with some also involving fibrous tissue; mixed echogenicity lesions were associated with the triphasic components; and hypoechoic lesions were mainly associated with primitive mesenchymal tissue. In case 6, a “fence-like pattern” corresponded to the alternating presence of adipose tissue and primitive mesenchymal tissue; in case 7 and 14, a “serpentine pattern” was associated with the interweaving and tortuous distribution of the triphasic components (Table 4).

**Table 4:** Correlation between ultrasonographic findings and histopathological results in eight children.

Case	Echogenicity	Sonographic patterns	Possible corresponding histologic components	Supporting immunohistochemical findings
2	Hyperechoic	None	Predominantly mature adipose tissue and primitive mesenchymal tissue	S-100 (+); Vim (+); CD34(−)
3	Hyperechoic	None	Mixed triphasic components	S-100 (+); SMA (+); Vim (+); CD34(+)
4	Hyperechoic	None	Mixed triphasic components	S-100 (+); SMA (+); Vim (+); CD34(+)
6	Heterogeneous hyperechoic	Fence-like	Predominantly mature adipose tissue and primitive mesenchymal tissue	S-100 (+); SMA (−); Vim (+); CD34(+)
7	Mixed echoic	Serpentine	Mixed triphasic components	S-100 (+); SMA (+); Vim (+); CD34(+)
8	Hyperechoic	None	Mixed triphasic components	S-100 (+); SMA (+); Vim (+); CD34(+)
12	Hypoechoic	None	Predominantly fibrous tissue and primitive mesenchymal tissue	S-100 (−); SMA (+); Vim (+); CD34(+)
14	Heterogeneous hyperechoic	Serpentine	Mixed triphasic components	S-100 (+); SMA (+); Vim (+); CD34(+)

S-100, S-100 protein; SMA, smooth muscle actin; Vim, vimentin; CD34, cluster of differentiation 34.

### 3.6 Analysis of potential factors associated with misdiagnosis

All 16 children were initially misdiagnosed on preoperative ultrasonography. Lipoma was the most common initial diagnosis (8/16, 50%), followed by infantile hemangioma (3/16, 18.75%). The remaining cases were misinterpreted as breast bud development, hernia, inflammatory mass, skin thickening, or malignant tumor, each accounting for one case.

A retrospective review suggested that the initial misdiagnosis was mainly related to the overlap between the ultrasonographic appearance of FHI and other superficial soft-tissue masses. Lesions initially diagnosed as lipoma were typically hyperechoic or slightly hyperechoic with a heterogeneous internal echotexture, and most showed sparse internal vascularity on CDFI. These ultrasonographic features closely resembled those of lipoma. Lesions misinterpreted as infantile hemangioma were generally slightly hyperechoic or mixed-echo masses with detectable but poor blood-flow signals. A superficial lesion in the subcutaneous fat layer of the left breast in a male infant was initially considered for breast bud development because of its superficial location, relatively homogeneous hypoechoic appearance, and scant vascularity. A right scrotal lesion with heterogeneous hyperechoic echotexture, well-defined margin, and regular morphology was interpreted as hernia because of suspected communication with the abdominal cavity on ultrasonography. A right axillary lesion with mixed echogenicity, indistinct margin, irregular morphology, and linear hypoechoic strands within hyperechoic areas was initially interpreted as an inflammatory mass. One superficial lesion confined to the perineal skin layer, with low echogenicity and scant vascularity, was misinterpreted as skin thickening. In eight lesions, the “fence-like pattern” or “serpentine pattern” was not recognized as a clue to FHI in the initial reports.

CT/MRI examinations were performed in 13 children for further lesion characterization and preoperative assessment after ultrasonography. Cross-sectional imaging findings failed to suggest FHI and were discordant with the ultrasonographic diagnoses in all 13 cases. Twelve cases were interpreted as benign, whereas one MRI examination showed no definite abnormality. Retrospectively, imaging findings were heterogeneous. Lesions with irregular or patchy soft-tissue density, internal septa or linear low-density area, low signal intensity on T1, high signal intensity on T2, and variable enhancement partially reflected the triphasic histologic composition of FHI; however, these findings were not sufficiently specific to avoid misdiagnosis. Among the six lesions with a “serpentine pattern”, CT/MRI mainly demonstrated heterogeneous signal intensity, internal septa, and uneven enhancement. The single lesion with a “fence-like pattern” that underwent MRI showed no definite abnormality.

### 3.7 A representative case misdiagnosed as malignant tumor

Case 16 was of a 19-month-old girl who presented with two palpable subcutaneous masses located on the right upper arm and distal right clavicular region. Both lesions were mobile, firm, and well circumscribed on palpation. Hair growth was observed on the skin surface overlying the right upper-arm lesion. Preoperative ultrasonographic examination revealed a heterogeneous, slightly hyperechoic mass, measuring 41 mm × 35 mm × 19 mm within the subcutaneous fat layer of the right upper arm, with relatively clear margin but irregular morphology. Scattered slit-like hypoechoic areas in the mass that alternated with hyperechoic areas were also observed, and these were distributed perpendicularly to the skin, similar to a “fence-like pattern”. Another heterogeneous, slightly hypoechoic lesion, measuring 13 mm × 10 mm × 5 mm was detected in the subcutaneous tissue near the distal right clavicle. CDFI revealed sparse punctate and linear vascular signals, and ultrasonographic elastography revealed that the texture of both lesions was relatively firm. Based on these findings, a malignant soft-tissue tumor was suspected. Case 16 did not undergo preoperative CT or MRI examination. Surgical excision was performed subsequently. The masses showed indistinct margin and lobulated morphology intraoperatively, with extension into the deep fascia but no muscular invasion. The intraoperative diagnosis favored lipoblastoma. Histopathological examination revealed a poorly circumscribed subcutaneous lesion that was composed of dense fibrous, primitive mesenchymal, and mature adipose tissues in varying proportion, all of which were accompanied by proliferating vessels. FHI was ultimately diagnosed (Figure 1).

The preoperative misdiagnosis was chiefly attributed to the heterogeneous echogenicity, irregular morphology, internal vascularity, and relatively firm elasticity of the lesion on ultrasonographic imaging, which raised the suspicion of a malignant soft-tissue tumor.

## 4 Discussion

FHI is a superficial soft-tissue tumor located within the dermis or subcutaneous tissue. FHI was initially reported by Reye (6) in 1956 and designated as “infantile subdermal fibromatosis”; in 1965, it was redefined by Enzinger (7). More than 200 cases of FHI have been reported worldwide (3), and although its onset and development have not been fully elucidated, Park et al. (8) found that epidermal growth factor receptor (EGFR) exon 20 insertion/duplication mutations constitute a major element in its pathogenesis.

FHI is commonly observed in infants and children under 2 years of age, with approximately 91% occurring within the first year of life. Approximately 15%–25% of cases are detected at birth, with some incidence occasionally found in older children (1). FHI is common in boys, with a male-to-female ratio of approximately 2.0–2.4:1. FHI can occur in various anatomical locations, manifesting most frequently in the axilla, upper limb, shoulder, thigh, inguinal region, upper trunk, back, and sacrococcygeal region (9, 10). In our study, 15 children (15/16, 93.75%) were under 2 years of age, and the male-to-female ratio was approximately 3:1. The most common site of occurrence was the axilla, followed by the scrotum, upper arm, chest wall, neck, and buttock—consistent with previous research findings (11).

The clinical manifestations of FHI are nonspecific. Most patients present with painless masses in the dermis or subcutaneous tissue with a tough texture and moderate mobility. A single lesion is common, whereas a few cases have multiple lesions (12). The size of the mass varies and generally ranges from 4 to 170 mm (with an average of 30 mm) (11), with rare giant lesions of up to 450 mm reported (13). Although the skin color of the mass typically appears normal, some patients may show hyperpigmentation, hypertrichosis, or hyperhidrosis, contributing to the diagnosis (1, 14). All children in our study presented with painless local subcutaneous masses and normal skin color. In addition, case 16 presented with two masses, one of which showed localized hair growth, which is rare.

The definitive diagnosis of FHI relies primarily on histopathological examination. FHI is histologically characterized by a distinctive triphasic pattern that encompasses the following admixed components in variable proportions: mature adipose tissue, intersecting fibrous collagen tissue, and primitive mesenchymal tissue rich in mucoid matrix (11). Although these components display clear boundaries, there can be a transition between the fibrous collagen tissue and the islands of primitive mesenchymal cells. The lesions may exhibit various degrees of diffuse fibrosis (2).

Previous studies have suggested a close correlation between ultrasonographic features of the FHI and its histopathological composition. For example, the FHI typically appears hyperechoic when fat and fibrous tissues are predominant. In contrast, the FHI may present as a hypoechoic or mixed cystic-solid echogenic structure when abundant primitive mesenchymal tissue is present, and fibrous tissue may result in increased stiffness on elastography (10, 15). Several studies have described two potentially suggestive ultrasonographic features in FHI, namely the “serpentine pattern” (i.e., interwoven hyperechoic and hypoechoic areas that follow a tortuous course) and the “fence-like pattern” where alternating hyperechoic and hypoechoic areas arise perpendicular to the skin (2, 15, 16). With respect to the etiology of the “serpentine pattern”, Arioni et al. (17) proposed that the hyperechoic areas within FHI lesions resulted from the intersecting arrangement of internal structures that created numerous acoustic interfaces; whereas Lee et al. (18) conversely postulated that the hyperechogenicity may have originated from the fatty components within the lesion, whereas the hypoechogenicity stemmed from its fibrous components. Nash et al. (19) proposed that skin ligaments (fibrous septa) connecting the dermis to the deep fascia were distributed throughout the subcutaneous fat. The fibrous septa may restrict lesion expansion and direct growth perpendicular to the skin surface, thereby contributing to the formation of the “fence-like pattern”. Primitive mesenchymal tissue, fat tissue, and possibly dilated vessels may also be included in hypoechoic areas within the “fence-like pattern” (2). In the present study, 12 lesions were mainly hyperechoic, three were hypoechoic, and two were mixed echoic. Among the former, six exhibited a “serpentine pattern” and two presented a “fence-like pattern”. A retrospective qualitative analysis suggested that these ultrasonographic manifestations may reflect a heterogeneous internal composition. However, histologic component proportions were not quantitatively assessed and immunohistochemical data were incomplete; therefore, these observations should be interpreted cautiously. Based upon both the extant literature and the findings of this study, the “serpentine pattern” and “fence-like pattern” may be suggestive—but not specific—ultrasonographic manifestations of FHI and should be interpreted together with clinical presentation and other imaging findings.

Importantly, the complex compositional structure of the FHI leads to its heterogeneous imaging appearance (which can be highly similar to that of other benign or malignant soft-tissue lesions), resulting in frequent misdiagnosis. In our cohort, lipoma was the most common misdiagnosis, accounting for 50% of all cases. We attribute this to the predominance of hyperechoic components within numerous lesions, reflecting abundant adipose tissue. In addition, the relatively sparse vascularity observed in our children further reinforced the impression of a benign fatty tumor. Infantile hemangioma was the second most commonly misdiagnosed condition, principally because of the presence of detectable internal vascular signals on CDFI. These findings suggest that even limited vascularity within the FHI can be interpreted as indicative of a vascular tumor. Other misdiagnoses, including breast bud development, hernia, inflammatory mass, skin-related condition, and malignant tumor, were associated with specific sonographic features, such as ill-defined margin, irregular morphology, heterogeneous echogenicity, and increased stiffness. One case in our series was notably misinterpreted as a malignant tumor due to its heterogeneous echogenicity, irregular morphology, and relatively firm elasticity, underscoring the potential of FHI to mimic aggressive lesion.

Among the children who underwent multimodal imaging evaluation in this cohort, the preoperative diagnostic impressions differed between ultrasonography and CT/MRI. This discrepancy likely reflects both the rarity of FHI and the heterogeneity of its imaging manifestations, as different imaging modalities provide complementary but distinct diagnostic information and may therefore lead to differing differential diagnostic considerations. Ultrasonography primarily depicts the lesion echogenicity, internal architecture, vascularity, and tissue stiffness. In contrast, CT and MRI emphasize the lesion extent, tissue composition, attenuation or signal characteristic, internal septation, and enhancement pattern.

Previous studies have reported that bands of fatty or fibrous tissue can be observed extending across lesions on CT and MRI and, in some cases, a characteristic parallel or whorled distribution may be visible. Adipose elements on MRI show high signal intensity on both T1- and T2-weighted images and signal suppression on fat-suppressed sequences with irregular peripheral enhancement (9, 15). However, these imaging features are not specific, and may overlap with those of other soft-tissue tumors. In the current analysis, 13 children underwent CT/MRI examinations. Most lesions were located within the dermis or subcutaneous fat layer and frequently demonstrated heterogeneous soft-tissue signal intensity, internal septa or linear low-density area, and variable enhancement patterns. These features have resulted in alternative interpretations, such as venous malformation, lymphatic malformation, infantile hemangioma, fibroma, and lipoblastoma, rather than FHI. Our findings suggest that no single imaging modality is sufficient for reliable preoperative diagnosis of FHI. Instead, accurate recognition may rely on the integrated interpretation of ultrasonographic and CT/MRI findings, together with clinical characteristics and awareness of the characteristic triphasic histopathological architecture of FHI. Notably, case 16 did not undergo preoperative CT/MRI examination despite ultrasonographic suspicion of malignancy. As the medical records provided no documented explanation for this management decision, no further analysis was conducted.

**Figure 2:**
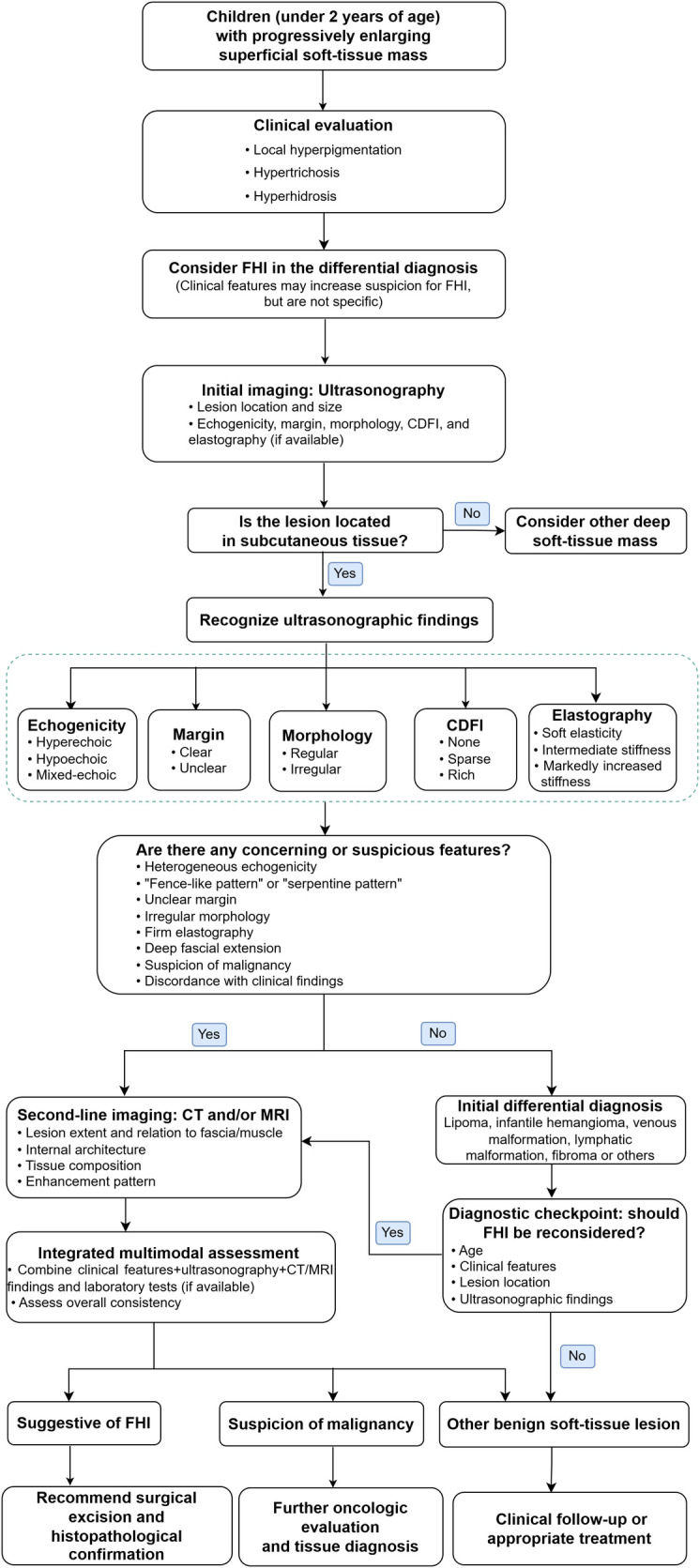
Proposed diagnostic flowchart for fibrous hamartoma of infancy (FHI) in children under 2 years of age.

Chen et al. (2) reported 28 cases of FHI in children, all of which were misdiagnosed on ultrasonograms. Similarly, all cases in our study were initially misinterpreted on preoperative ultrasonography, highlighting the high misdiagnosis rate and the diagnostic challenges of this disease. Therefore, enhancing the awareness of this disease among pediatricians and ultrasonographers is crucial for reducing misdiagnoses, improving the accuracy of initial diagnoses, and facilitating early surgical interventions. Based on our analysis of the misdiagnosed cases in this study, and in conjunction with findings from the existing literature, we propose a diagnostic flowchart for reducing the misdiagnosis of FHI (Figure 2). The flowchart emphasizes a stepwise and integrative multimodal approach combining clinical characteristics, ultrasonography, and CT/MRI evaluation to reduce misdiagnosis and improve the preoperative recognition of FHI. Histopathological examination is essential for a final diagnosis.

## 5 Limitations

The current study has several limitations. First, this was a retrospective single-center study with a relatively small-sample size owing to the rarity of FHI, which may introduce a selection bias and limit the generalizability of our findings. Second, the ultrasonographic equipment and examination protocols were not fully standardized across the study period, and not all children underwent comprehensive multimodal imaging evaluation, limiting further comparisons among imaging modalities. Third, the ultrasound–histopathology correlation analysis was qualitative and could not be formally validated in all cases. Finally, the proposed diagnostic flowchart was developed from a small retrospective case series and has not been externally validated, its diagnostic performance and clinical applicability should be interpreted with caution. Therefore, future multicenter studies with standardized imaging and prospective validation are warranted.

## 6 Conclusions

FHI is a relatively rare, benign, superficial soft-tissue tumor. Owing to its nonspecific clinical manifestations and variable imaging features, FHI is frequently misdiagnosed prior to surgery. In this study, all cases were initially misdiagnosed on ultrasonography, and the preoperative diagnoses differed from those based on CT/MRI. The “serpentine pattern” and “fence-like pattern” may be suggestive—but not specific—ultrasonographic manifestations of FHI. The proposed diagnostic flowchart, which emphasizes integrative interpretation of clinical characteristics and multimodal imaging findings, may improve diagnostic accuracy and reduce the misdiagnosis rate of FHI. Nevertheless, a definitive diagnosis relies on histopathological examination.

## Data Availability

The original contributions presented in the study are included in the article/Supplementary Material, further inquiries can be directed to the corresponding author.
